# Supplementary material for: Clinical prediction rule for SARS-CoV-2 infection from 116 U.S. emergency departments 2-22-2021
Source: PLoS One. 2021 Mar 10;16(3):e0248438. doi: 10.1371/journal.pone.0248438 (PMC7946184; doi:10.1371/journal.pone.0248438)
Supplement: S1 Table — (DOCX) [file pone.0248438.s001.docx]

Supplemental Table

| **Table S1**. P values from unpaired t-test or Chi-Square |  |
| --- | --- |
| Predictor | P (SARS-CoV-2+ versus SARs-CoV-2-) |
| Edoxaban taken prescribed prior to qualifying SARS-CoV-2 test | N/A |
| Vomiting / Nausea prior to SARS-COV-2 testing | 0.98 |
| Dabigatran taken prescribed prior to qualifying SARS-CoV-2 test | 0.93 |
| Race - Native Hawaiian or Other Pacific Islander | 0.87 |
| Altered mental status/confusion prior to SARS-COV-2 testing | 0.72 |
| Arrival to the emergency department by an emergency medical service (EMS) vehicle | 0.6 |
| Shortness of breath (Dyspnea)recorded during index ED visit | 0.56 |
| Bloody sputum/haemoptysis recorded during index ED visit | 0.54 |
| Aspirin taken within 24 hours of index visit | 0.5 |
| Unable to collect substance abuse data per institution | 0.42 |
| Risk for COVID-19 infection - Institutional exposure:  prison/jail or other correctional facility | 0.41 |
| Skin ulcers prior to SARS-COV-2 testing | 0.39 |
| Cough with sputum production (wet) recorded during index ED visit | 0.3 |
| Oropharynx bleeding (when “Bleeding (Haemorrhage)” was selected as a symptom prior to SARS-COV-2 testing) | 0.26 |
| Ibuprofen taken within 24 hours of index visit | 0.24 |
| Joint pain (Arthralgia) recorded during index ED visit | 0.24 |
| Risk for COVID-19 infection - Group home | 0.24 |
| Nose bleeding (when “Bleeding (Haemorrhage)” was selected as a symptom prior to SARS-COV-2 testing) | 0.23 |
| Cutaneous bleeding (includes easy bruising) (when “Bleeding (Haemorrhage)” was selected as a symptom prior to SARS-COV-2 testing) | 0.19 |
| Low molecular weight heparin taken prescribed prior to qualifying SARS-CoV-2 test | 0.15 |
| Acetaminophen taken within 24 hours of index visit | 0.14 |
| Ear pain recorded during index ED visit | 0.11 |
| Ever hypotensive in ED (SBP < 100 mm Hg) | 0.1 |
| Risk for COVID-19 infection - Employed, healthcare worker with patient contact | 0.09 |
| Cardiac arrest prior to SARS-COV-2 testing | 0.08 |
| Heart Rate | 0.08 |
| Race - American Indian or Alaska Native | 0.08 |
| Currently pregnant | 0.2 |
| Vaginal bleeding (when “Bleeding (Haemorrhage)” was selected as a symptom prior to SARS-COV-2 testing) | 0.06 |
| Syncope prior to SARS-COV-2 testing | 0.06 |
| Warfarin taken prescribed prior to qualifying SARS-CoV-2 test | 0.05 |
| Conjunctivitis prior to SARS-COV-2 testing | 0.03 |
| Lymphadenopathy prior to SARS-COV-2 testing | 0.03 |
| Risk for COVID-19 infection - Travel to the USA from a country with known endemic disease | 0.02 |
| Lowest O2 saturation while in ED | 0.02 |
| Risk for COVID-19 infection - Institutional exposure: assisted living facility | 0.02 |
| Abdominal pain | <0.0001 |
| Ace inhibitors taken within 24 hours of index visit | <0.0001 |
| Age at time of visit | <0.0001 |
| Angiotensin receptor blocker use | <0.0001 |
| Apixaban taken prescribed prior to qualifying SARS-CoV-2 test | <0.0001 |
| Asthma (prior history) | <0.0001 |
| Atrial fibrillation (prior history or new onset) | <0.0001 |
| BiologicalSex | <0.0001 |
| Bleeding (Haemorrhage)prior to SARS-COV-2 testing | <0.0001 |
| Bloody emesis (when “Bleeding (Haemorrhage)” was selected as a symptom prior to SARS-COV-2 testing) | <0.0001 |
| BMI | <0.0001 |
| Cancer (active or in remission) | <0.0001 |
| Chest pain recorded during index ED visit | <0.0001 |
| Chronic obstructive pulmonary disease (prior history) | <0.0001 |
| Cough without sputum (dry) recorded during index ED visit | <0.0001 |
| Daily alcohol use | <0.0001 |
| Diabetes Mellitus (prior history) | <0.0001 |
| Diarrhea prior to SARS-COV-2 testing | <0.0001 |
| Diastolic Blood Pressure (mm Hg) | <0.0001 |
| Fatigue / Malaise recorded during index ED visit | <0.0001 |
| Headache prior to SARS-COV-2 testing | <0.0001 |
| Heart failure (either systolic or ejection fraction preserved) | <0.0001 |
| History of fever recorded during index ED visit | <0.0001 |
| Hyperlipidemias (hyperlipidemia of any type, includes currently taking a statin) | <0.0001 |
| Latin Ethnicity | <0.0001 |
| Marijuana use | <0.0001 |
| Medicaid or no insurance | <0.0001 |
| Methamphetamine use | <0.0001 |
| Muscle aches (Myalgia) recorded during index ED visit | <0.0001 |
| No anticoagulants taken prescribed prior to qualifying SARS-CoV-2 test | <0.0001 |
| No known risk for COVID-19 infection | <0.0001 |
| No substance use | <0.0001 |
| Obesity (by diagnosis, provider interpretation or body mass index >35 Kg/m^2) | <0.0001 |
| Olfactory/taste disturbance prior to SARS-COV-2 testing | <0.0001 |
| Opioid dependency | <0.0001 |
| Organ transplantation (any organ) | <0.0001 |
| Other (not specified) antihypertensive medication taken within 24 hours of index visit | <0.0001 |
| Other bleeding site (not specified) (when “Bleeding (Haemorrhage)” was selected as a symptom prior to SARS-COV-2 testing) | <0.0001 |
| Other lung disease (pulmonary fibrosis, cystic fibrosis, bronchiectasis, pulmonary hypertension) | <0.0001 |
| Cocaine use | <0.0001 |
| Injection drug use | <0.0001 |
| Oxygen saturation at triage | <0.0001 |
| Prior ischemic heart disease (myocardial infarction, or any coronary revascularization including stent or CABG) | <0.0001 |
| Prior venous thromboembolism | <0.0001 |
| Race - Asian | <0.0001 |
| Race - Black or African American | <0.0001 |
| Race - Unknown/Other | <0.0001 |
| Race - White | <0.0001 |
| Recent seizure prior to SARS-COV-2 testing | <0.0001 |
| Respiratory distress on arrival | <0.0001 |
| Respiratory Rate (breaths/min) | <0.0001 |
| Risk for COVID-19 infection - Caretaker, partner or family member in close contact with a person with known or suspected COVID-19 | <0.0001 |
| Risk for COVID-19 infection - Employed, non-health care worker, contact with family, and/or friends, and/or general public | <0.0001 |
| Risk for COVID-19 infection - Institutional exposure:, nursing home | <0.0001 |
| Risk for COVID-19 infection - Other risk exposure (not specified) | <0.0001 |
| Risk for COVID-19 infection - Person experiencing homelessness | <0.0001 |
| Risk for COVID-19 infection - Sick contacts without confirmed COVID-19 | <0.0001 |
| Risk for COVID-19 infection - Unemployed or retired, social contact with friends, family and/or general public | <0.0001 |
| Rivaroxaban taken prescribed prior to qualifying SARS-CoV-2 test | <0.0001 |
| Runny nose (Rhinorrhoea) recorded during index ED visit | <0.0001 |
| Skin rash | <0.0001 |
| Smoker (Current smoker regardless of number of cigarettes; includes vaping, cigars or pipe smoking) | <0.0001 |
| Sore throat recorded during index ED visit | <0.0001 |
| Statins taken within 24 hours of index visit | <0.0001 |
| Systemic Hypertension (Regardless of treatment status; can also be diagnosed by diastolic BP 110 mm Hg in ED) | <0.0001 |
| Systolic Blood Pressure (mmHg) | <0.0001 |
| Temperature (°C) | <0.0001 |
| Thiazolidinediones taken within 24 hours of index visit | <0.0001 |
| Total number of symptoms | <0.0001 |
| Wheezing recorded during index ED visit | <0.0001 |
