# Supplementary material for: Clinical prediction rule for SARS-CoV-2 infection from 116 U.S. emergency departments 2-22-2021
Source: PLoS One. 2021 Mar 10;16(3):e0248438. doi: 10.1371/journal.pone.0248438 (PMC7946184; doi:10.1371/journal.pone.0248438)
Supplement: Attachment — Submitted filename: Response to reviewers CORC at PLOS ONE v3.docx [file pone.0248438.s003.docx]

ONE-D-21-00785

Clinical prediction rule for SARS-CoV-2 infection from 116 U.S. emergency departments

PLOS ONE

**We thank the reviewers for their excellent and detailed reviews. We have responded to each point completely, including the addition of new data where requested. All authors have reviewed and approved these responses and the revised manuscript. Our responses are in bold font. To reduce the reviewers’ workload, we have pasted the changes or additions into this response letter in quotations and identified the section and paragraph in the revised manuscript where these changes can be found. Also, we read and made the changes to author information/affiliations and formatting per the links from editorial staff below.**

**We hope these extensive and careful revisions meet with full acceptance.**

**Jeffrey A. Kline on behalf of all authors.**

When submitting your revision, we need you to address these additional requirements.

1. Please ensure that your manuscript meets PLOS ONE's style requirements, including those for file naming. The PLOS ONE style templates can be found at

https://journals.plos.org/plosone/s/file?id=wjVg/PLOSOne_formatting_sample_main_body.pdf and

https://journals.plos.org/plosone/s/file?id=ba62/PLOSOne_formatting_sample_title_authors_affiliations.pdf

**We have made these stylistic changes as requested.**

2. Please provide additional details regarding participant consent.

If you are reporting a retrospective study of medical records or archived samples, please ensure that you have discussed whether all data were fully anonymized before you accessed them and/or whether the IRB or ethics committee waived the requirement for informed consent. If patients provided informed written consent to have data from their medical records used in research, please include this information.

Once you have amended this/these statement(s) in the Methods section of the manuscript, please add the same text to the “Ethics Statement” field of the submission form (via “Edit Submission”).

**Done as requested**

Reviewer #1:

Thank you for the opportunity to review this manuscript which describes the development and validation of the (COVID Rule Out Criteria) CORC models for predicting a PCR positive infection with COVID-19 among patients presenting to a large network of US emergency departments. Overall, I am very impressed with this project and identify a number of strengths. Firstly the authors are to be commended on the robustness of the data set as well as the promptness for achieving data collection analysis and reporting within 10 months of the start of the pandemic. The project also benefits from adherance to the tripod reporting guidelines as well as a robust methodology for high quality data collection end a fairly clear approach to the development of both the simplified and more comprehensive CORC rule. Also commendable are the emphasis on readily collected clinical data points that bodes well for this rules uptake into clinical practice. Lastly on the list of kudos as a emergency physician who who has provided care for hundreds of suspected COVID-19 patients the CORC model has significant face validity and jives with what corresponds to both my clinical impression as well as the literature to date.

I do have a few questions and suggestions for the authors to consider. I wonder if an additional justification for the use of the CRC score or rule could relate to the limited performance characteristics of PCR testing. Specifically, if the PCR test have some optimal specificity and can result in false positives from remote and no longer active infections would the CORC be another justification four streamlining testing and not providing it widely in ED settings.

**Authors appreciate the endorsement. Thank you for this recommendation. We have added the following line to the end of the third paragraph in the discussion:**

“After prospective validation, the CORC score may also help reduce low-value repeated molecular testing weeks or months after initial infection that could produce false positive results.”

I also wish that the authors can help elaborate on why smoking appears to be protected in the CORC model. Additionally, it is curious to me that dyspnea was not an independent predictor variable and a better explanation of this would be appreciated.

**We will soon submit a paper specifically addressing this finding in more detail. For this paper, we do mention that this finding has been previously reported and we do cite a paper that poses the hypothesis that nicotine reduces epithelial expression of the ACE2 receptor as a potential mechanism. We have added the following modification to the end of the fifth paragraph in the Discussion. Dyspnea in an of itself did not increase risk of testing positive.**

“A potentially unanticipated finding is that smoking history was retained as a negative predictor of infection—a finding that has been reported by others who have suggested that nicotine may reduce expression of epithelial ACE2 receptor, and thus reduce SARS-CoV-2 infectivity.^22-24^”

Similarly the absence of gastrointestinal symptoms is interest because at the present time the emergency Department where I work will subject patients with those complaints two isolation procedures. I presume therefore that the failure of nausea and vomiting diarrhea to be included in the CORC model is it reflection off it's insignificant contribution to predicting COVID-19 infection.

**Correct, while GI symptoms were ultimately “statistically significant” as predictors in the forward stepwise model, they did not carry sufficient weight to include in the rule.**

I think it is important for the authors to provide more clarity on the ethnic associations with COVID-19 infection in order to make the project more generalizable. The key question is whether being black or of Hispanic origin is somehow a genetic predisposition to infection or if from a social determinants of health perspective these groups are more likely to be frontline workers and live in communal settings that places them at risk.

**We appreciate this comment and specifically addressed it in an entire free-standing paragraph in the Discussion:**

“Compared with persons identifying as White, Black race and Latino/Hispanic ethnicity significantly increased the probability of infection. Race-specific patterns in symptom manifestation that might alter clinical suspicion and testing threshold do not appear to explain the differences in positive rate.^25^ To our knowledge, no genetic or biologic reasons explain why Black and Hispanic/Latino patients are more likely to have a SARS-CoV-2 infection. Instead, the statistical weight on these variables may result from them acting as proxies for other societal factors. The association of race with positive testing may correlate with a higher likelihood of working service-related jobs which are unable to be done from home (thereby increasing exposure to SARS-CoV-2). In one study of a cohort of SARS-CoV-2 infected patients in Louisiana, 77% of those requiring hospitalization were Black; only 30% of the total area population is Black.^26^ However, when adjusted for socioeconomic status and pre-existing clinical comorbidities, there was no racial difference identified in mortality.^26^ Ongoing work will report the impact of insurance status and geographic location (by four digit zip code) on SARS-CoV-2 infection rate and severity.”

Final point on application of a validated model relates to the potential use of the CORC in a shared decision-making context. Patients present to the emergency Department with an expectation or desire to undergo COVID-19 testing and in theory at least the CRC model can't present an actual risk that can be discussed in the context of the potential downsides of undergoing testing.

**We agree that much work will need to be done to test the acceptability of the rule--perhaps instead of a molecular test.**

In summary this is obviously to me a high impact publication and the models that have been developed should be prioritized for urgent validation. This work should be disseminated promptly so that other groups around the world who are engaged in similar research can have the opportunity to validate the model in their setting.

**We appreciate the reviewer’s careful consideration.**

Reviewer #2:

Summary

The authors used registry data from the RECOVER network to build a predictor that predicts whether a patient was tested positive or negative for Covid-19. To this end, they used a stepwise forward logistic regression to reduce the number of independent variables to 13. Based on these 13 independent variables, they built a CORC rule that assigns a point system that can be used to decide the likelihood of the patient to have Covid-19. It was found that those with >5 CORC rule (maximum possible score 13) had >75% probability of infection. Although a practical CORC rule that can easily be adapted in the field was given by the authors, the manuscript lacks comparison with other machine learning methods and visual illustration (figures, tables) that helps the reader understand the work.

Major

- Have you tried other machine learning methods? How does the logistic regression compare to the other models like decision tree, SVM, neural network, etc.? The authors state the reason why they chose logistic regression amongst other models in the discussion. But it would be helpful for the readers to show the actual results in the manuscript.

**We would first note that to the best of our knowledge, this is the first model that does not include other laboratory and radiographic variables, this making this model useful in many high throughput settings, such as the triage area of emergency departments. To emphasize this, we have added this line to the first paragraph of the discussion:**

“To our knowledge, this is the first prediction rule for SARS-CoV-2 infection that does not require laboratory or radiographic data which makes this model very useful in high throughput settings, such as triage areas of emergency departments and also in non hospital settings such as homeless encampments and street medicine.”

**Authors of this work did use machine learning in a published paper, cited in this work. We have now added the AUC data to the limitations paragraph of the discussion:**

“Additionally, it remains possible that machine-based learning methods may offer a superior role, although as a preliminary step, several of the authors of this work, directly compared three derivation techniques (logistic regression, random forest and gradient boosting) to create prediction models for SARS-CoV-2 using ED-based data. The logistic regression model had an AUC of 0.89 (95% confidence interval [CI] = 0.84 to 0.94); the random forest method had AUC of 0.86 (95% CI = 0.79 to 0.92) and gradient boosting had an AUC of 0.85 (95% CI = 0.79 to 0.91).^28^ It is important to note that all prior prediction models included use of laboratory and radiographic values.”

- It’s difficult to understand the landscape of the data used in the manuscript. For example, how many features (independent variables) are there in total? How many of them are categorical variables and how many of them are non-categorical variables?

**We realize that our supplemental table was not accessible, and apologize for this oversite. We hope it is uploaded this time. To ensure ease of seeing it, we appended the table to this review and highlighted the parametric values. Of the initial 107 variables, 10 were parametric and 97 were dichotomous.**

- How does the logistic regression model differ from the CORC rule and the CORC score? What is the objective of each system?

**We recognize these terms sound similar, but to clarify, in the previous version the CORC score was the P value from the antilog of the logistic regression equation and the CORC rule was the simplified numerical point system derived from the coefficients of the logistic regression equaiotin. The logistic regression was the quantitative means to get to the CORC rule and provide coefficients to estimate the numerical values of each of the 13 questions in the CORC rule. We agree this was confusing.**

****To address this confusion, in the revision we have changed the nomenclature to speak of the “Probability from logistic regression” to replace the old term of “CORC score” and we now refer to the simplified rule presented in Table 3 as the “CORC score”. The term CORC rule is now gone from the paper.**

- The manuscript needs to report the statistical results for different conditions using tables. For example, what is the confusion matrix for the final logistic regression model built using 13 independent variables? It should also include complete statistics like precision, recall, F1, etc. Similarly, figures that show the precision-recall curve and ROC curve should be added.

**To address these important requests, we have added the following text and table to new paragraph four of the results:**

“Table 4 shows the standard diagnostic contingency table (sometimes referred to as a confusion matrix) using a CORC score >0 as the definition of a positive test result with associated calculations of precision, recall and F1 index. **Fig 2** shows the plots of the precision-recall curve and receiver operating characteristic curve with their AUC data. The CORC rule had a slightly lower area under the curve (0.75, 0.74-0.76) than the CORC score (0.80, 0.79-0.81)”

| Table 4. Contingency table (confusion matrix) for the CORC score | | |
| --- | --- | --- |
|  | CORC>0 | CORC<=0 |
| SARS-CoV-2+ | 3271 | 151 |
| SARS-CoV-2- | 5201 | 1302 |
| Precision = 3271/(3272+5201) = 0.39 | | |
| Recall =3271/(3271+151 ) = 0.96 | | |
| F1 = (0.39*0.96)/(0.39+0.96) = 0.55 | | |
| Abbreviations: CORC-COVID-19 Rule-Out Criteria | | |

**Fig 2. Diagnostic performance of the CORC score.** The plot in the top panel shows the precision-recall curve and the plot in the lower panel shows the receiver operating characteristic curve for the COVID-19 Rule Out Criteria (CORC) score.

- How does the performance change as the number of selected features (variables) decreases while performing stepwise logistic regression? And what was the ‘quantitative’ reason for selecting the 13 variables? If we select 15 variables instead of these 13, for example, will we have better performance?

**The reason for the 13 variables is this was the point of best fit by looking for the bottom of the Akaiki information index. Also, more variables would cause the model to lose sensibility and real-world utility. We have added this line to the statistics section:**

“The model was terminated when the change Akaiki information criterion (AIC=2k-2ln(L) where k=number of variables and L=maximum likelihood) reached its nadir.”

Medium

- I have difficulty understanding the difference between the data obtained through RECOVER (3rd paragraph of the Methods section) and 28 symptoms (4th paragraph of the Methods section). The manuscript makes it think that these are all different sources of data. Also, the authors mention ‘form’. What is this form? It wasn’t clearly defined previously.

**All data came from the medical records of patients who presented for care at the emergency departments at the sites representing the RECOVER network. The 28 symptoms were extracted from the medical records and entered into a REDcap form, which is the form being mentioned.**

**We have added the word REDCap in front of the word form throughout to help clarify this issue, and revised the paragraph about the 28 symptoms as follows:**

“To generate a comprehensive pool of independent predictors for a quantitative pretest probability model, as well as harmonization with other data, among the 204 questions, we recorded 28 symptoms, including all symptoms from the Clinical Characterization Protocol from the World Health Organization-supported International Severe Acute Respiratory and Emerging Infection Consortium (ISARIC).^13^”

- What is the difference between the 30% criterion standard positive rate and the 50% random sampling? Terminologies are not clearly defined here. It would help to have a table that shows how the data was split.

**The 30% refers to patients with a positive molecular test for SARS-CoV-2 and the 50% represents the derivation and validation sets. The characteristics of patients representing these two 50% samples is shown in Table 1.**

- ‘Variables with P<0.05 were entered into logistic regression equation…’ What are these variables? How do these variables differ from the variables selected for rule development?

**These are shown in S1 Table 1, which is now appended to this response and is uploaded on the PLOS ONE submission site.**

- Why is it important to retain the performance of the CORC from the earlier in the year to the later?

**The demographics of the case mix and severity may be changing. For example, communities with more dense social living conditions may have been hit harder, earlier, and disease severity may have been greater early on. The latter can produce spectrum bias.**

- Can you build a better CORC rule by assigning different points to each feature? For example, Assign a higher point for ‘loss of sense of taste or smell’ than the race?

**Authors believe is a very good question, and the answer is that using the numbers exactly proportional to the coefficients from the logistic did not increase the AUC on the ROC for the CORC rule. Also, as the author team is largely emergency physicians, they are well aware of the extreme need to keep rules as simple as possible. This has been shown with previous rules, including the Wells’ score for pulmonary embolism.**

- I could not located the supplemental table 1. The link to the supplemental information directed me to the Kline et al. 'Multicenter registry of United States emergency department

patients tested for SARS-CoV-2'.

**We apologize for the inconvenience. The Table has been uploaded as a supplement. To be certain, it is appended to this response letter.**

Minor

- The authors state in the introduction ‘In 2016, the >5000 US EDs had approximately 145 million patients.’ Are there more recent statistics after the covid-19 pandemic?

**Not as of yet. The current common belief is that the number will decrease.**

- Authors use the term predictor and variable interchangeably. I suggest using ‘variable’ to be consistent and reduce confusion among the readers.

**Done**

Reviewer #3:

SUMMARY: The manuscript describes logistic regression-based model that can identify the probability of a positive SARS-CoV-2 test using most accessible clinical features from patients in the US. The manuscript is well written, the data appears to be of high value, the data analysis methodology is appropriate although too simple, and the results are valuable. I have several suggestions that if not addressed can substantially undermine the potential impact of the manuscript.

Note, I did not find the supplemental Table 1.

**We apologize for the inconvenience, and have made sure it is uploaded, and also appended it to this response letter**

************************

MAJOR ISSUES

1) Impact of testing method: Given the variable accuracy of SARS-CoV-2 test methods (using swab or blood samples), it is important to analyze and report whether the predictive model is the same, or different regardless of the testing method. Once done, It will be important to emphasize the predictive model that is only trained on a portion of data that uses the more reliable testing method.

**The model is based upon a criterion standard of any positive test for SARS-CoV-2 within 30 days. The vast majority of tests were nasal swabs. Only 3% of patients had serology done, and very few were positive. We do present data about the model performance in patients who initially tested negative and then later tested positive:**

“From the entire sample (both derivation and validation), the initial swab that qualified the patient for enrollment was negative in 13,159 patients. Of these, 174 (1.1%) subsequently had evidence of SARs-CoV-2 from either a repeated nasopharyngeal swab or positive antibody testing done within 30 days. Among these 174 patients who had a possibly false negative molecular test done on a swab sample, the CORC rule was >0 in 87%.”

2) Comparison with baseline: Authors mention previously published predictive models, however do not show any numerical comparison of such models in the context of their data. For specificity, sensitivity and area under the curve values, the baseline model numbers should also be mentioned (the baseline here would be a model that always predicts the test to be negative).

**We now provide comparison of AUC values in the limitations paragraph of the discussion:**

“Additionally, it remains possible that machine-based learning methods may offer a superior role, although as a preliminary step, several of the authors of this work, directly compared three derivation techniques (logistic regression, random forest and gradient boosting) to create prediction models for SARS-CoV-2 using ED-based data. The logistic regression model had an AUC of 0.89 (95% confidence interval [CI] = 0.84 to 0.94); the random forest method had AUC of 0.86 (95% CI = 0.79 to 0.92) and gradient boosting had an AUC of 0.85 (95% CI = 0.79 to 0.91).^28^ It is important to note that all prior prediction models included use of laboratory and radiographic values.”

3) Conclusions are not very actionable: For results to be more actionable, it would be very valuable to provide recommendations in the following form (or something similar, otherwise it seems very unfortunate that such conclusions cannot be made!): Patients with a CORC score above HH.H threshold can be considered COVID-19 positive with 95% confidence and patients with CORC score below LL.L threshold can be considered negative with 95% confidence. For other patients (i.e. CORC score between LL.LL and HH.HH) molecular testing is needed for a high confidence diagnosis. Upon medical resource limitations (timely molecular testing, treatment capacity), the CORC score can be used for testing and treatment prioritization of patients. In areas that molecular testing is unavailable, the prevalence of COVID-19 can be estimated using the average CORC score of patients to be interpreted using Table XX which shows the estimated positivity rate for different CORC scores.

**We agree completely and have now included the following statement in the second paragraph of the discussion:**

“Thus, assuming the expected prevalence of SARS-CoV-2 infection is below 10%, the estimated likelihood ratio negative of 0.22, a CORC rule of zero or less would allow a very low posterior probability (e.g., <2.0%) and thus may obviate the need for molecular testing or isolation in a negative pressure room in the ED setting. On the other hand, a CORC rule ≥5 should be considered high risk, suggesting the need for molecular testing, and possibly repeated testing if the first test is negative.^16^ “

**We would further offer that this statement puts the recommendation into perspective of the expected underlying prevalence. The prevalence of SARS-CoV-2 is steadily decreasing, and is or soon will be below 9% in the generic, undifferentiated emergency populations who walk in for care in any given ED. It appears that in some sites, even among those ED patients selected for testing based upon clinical suspicion of COVID, the test positive prevalence will soon be below 9%. As for data to back that assertion, we have performed a real time interrater reliability and preliminary validation study (data are new and unpublished) of the CORC rule in Indianapolis. We have enrolled 400 undifferentiated ED patients and found that 105 (26%) were tested for SARs-CoV-2, but only 10 (9.5%) were positive.**

4) Predictive modeling method is too simple: The predictive modelling approach is not wrong but seems too simple. More sophisticated methods such as artificial neural networks (ANN), random forests (RF) or decision tress (DT) that are capable of modeling multiplicative relationships between variables are advisable given that the number of features is low compared to number of records. A DT based model is particularly applicable since it can be directly used to extract rules instead of the point-based formula. I’d suggest to use a data-driven modelling approach (such as DT based) instead of the hand-made model, for providing simple rules since the techniques such as dichotomization are not recommended (see “Royston, P., Altman, D. G., & Sauerbrei, W. (2006). Dichotomizing continuous predictors in multiple regression: a bad idea. Statistics in medicine, 25(1), 127-141.”)

**We respectfully point out that the “old fashioned” stepwise method still has value and has produced some of the most robust and useful decision rules in medicine, especially when the goal is simplicity. DT methods cannot produce a simple numeric score-based approach, which is needed in the emergency care setting. And to use the words of Harvey Feinstein, sensibility (meaning the utility in real practice) is as important as the quantitative methods to derive the rule. The dichotomizations used in this work were based upon the midpoint of the difference in means (between + and -), which also corresponded to the optimal cutoff of the peak diagnostic odds ratio on ROC for age, SpO2% and temperature. But stating “the midpoint between means” is much easier to understand for most readers. We point out that authors of this work directly compared logistic methods to RF and did not find RF to be superior.**

************************

MINOR ISSUES:

1) Authors mention “All sites were contracted to abstract charts from at least 500 patients”. As a person who does not work in a hospital, I don’t understand this sentence.

**This was an effort to reduce bias in sampling, meaning an reasonably large minimum sample size from each of the sites, and to provide geographic and demographic diversity.**

2) Authors mention “The mean values from five iterations were used, and compared with the pre-imputation mean to confirm a significant change (P<0.05)”: I don’t understand this sentence.

**We have deleted this sentence, recognizing that it may be hard to understand unless the reader is well versed in imputation strategy. This is a standard step to test if the imputation replacements significantly changed the mean value of the sample.**

3) Authors mention “variables selected for rule development using an empirical stepwise forward approach using the likelihood ratio approach”, but I didn’t find the exact method and threshold used.

**We have added the following line to better explain**

“The model was terminated when the change Akaiki information criterion (AIC=2k-2ln(L) where k=number of variables and L=maximum likelihood) reached its nadir.”

4) Authors mention “values were missing in less than 1% of the sample”. Replace “sample” with “samples”.

**Corrected.**

5) Missing value imputation creates a potential bias. Therefore will be valuable to also evaluate the model on records that have no missing values for the features that are used by the model.

**We appreciate this point, but in the end, none of the variables used in the retained CORC score and rule had more than 1% missingness (as above), which seems to mitigate this concern.**

6) Authors mention “Compared with US Census Bureau data from 2019, this ED sample is older, has a higher representation of persons identifying as Black (and lower percentage of persons identifying as White”. It is valuable to put the relevant numerical values here for the reader to see.

**We appreciate this point and have added the data as requested:**

“Compared with US Census Bureau data from 2019, the median age of this ED sample is older by approximately 12 years, and has approximately a 13% higher representation of persons identifying as Black (and lower percentage of persons identifying as White ), but a similar distribution of biological sex and Hispanic or Latino ethnicity.^15^”

7) “probability (P)” is mentioned a few times without clarifying what it represents/means here. This maybe a first class term in the data analysis software used (SPSS) that readers may not be familiar with.

**We apologize for the deficiency. This is not an SPSS term. We considered this to be a relatively well recognized concept in logistic regression. We have added this further explanation of P to the analysis section:**

“To test for validity, the CORC score was computed by solving for probability (P) from the logistic regression equation (obtained from the antilog of the logistic regression equation yielding P=[1+exp(-Σcoefficients+intercept)]^-1^);”

6. PLOS authors have the option to publish the peer review history of their article (what does this mean?). If published, this will include your full peer review and any attached files.

**We do not believe this comment pertains to the authors.**

If you choose “no”, your identity will remain anonymous but your review may still be made public.

Do you want your identity to be public for this peer review? For information about this choice, including consent withdrawal, please see our Privacy Policy.

Reviewer #1: Yes: Eddy Lang

Reviewer #2: Yes: Jason Youn

Reviewer #3: No

[NOTE: If reviewer comments were submitted as an attachment file, they will be attached to this email and accessible via the submission site. Please log into your account, locate the manuscript record, and check for the action link "View Attachments". If this link does not appear, there are no attachment files.]

While revising your submission, please upload your figure files to the Preflight Analysis and Conversion Engine (PACE) digital diagnostic tool, https://pacev2.apexcovantage.com/. PACE helps ensure that figures meet PLOS requirements. To use PACE, you must first register as a user. Registration is free. Then, login and navigate to the UPLOAD tab, where you will find detailed instructions on how to use the tool. If you encounter any issues or have any questions when using PACE, please email PLOS at figures@plos.org. Please note that Supporting Information files do not need this step.

Supplemental Table

| **Table S1**. P values from unpaired t-test* or Chi-Square |  |
| --- | --- |
| Predictor variable | P (SARS-CoV-2+ versus SARs-CoV-2-) |
| Edoxaban taken prescribed prior to qualifying SARS-CoV-2 test | N/A |
| Vomiting / Nausea prior to SARS-COV-2 testing | 0.98 |
| Dabigatran taken prescribed prior to qualifying SARS-CoV-2 test | 0.93 |
| Race - Native Hawaiian or Other Pacific Islander | 0.87 |
| Altered mental status/confusion prior to SARS-COV-2 testing | 0.72 |
| Arrival to the emergency department by an emergency medical service (EMS) vehicle | 0.6 |
| Shortness of breath (Dyspnea)recorded during index ED visit | 0.56 |
| Bloody sputum/haemoptysis recorded during index ED visit | 0.54 |
| Aspirin taken within 24 hours of index visit | 0.5 |
| Unable to collect substance abuse data per institution | 0.42 |
| Risk for COVID-19 infection - Institutional exposure:  prison/jail or other correctional facility | 0.41 |
| Skin ulcers prior to SARS-COV-2 testing | 0.39 |
| Cough with sputum production (wet) recorded during index ED visit | 0.3 |
| Oropharynx bleeding (when “Bleeding (Haemorrhage)” was selected as a symptom prior to SARS-COV-2 testing) | 0.26 |
| Ibuprofen taken within 24 hours of index visit | 0.24 |
| Joint pain (Arthralgia) recorded during index ED visit | 0.24 |
| Risk for COVID-19 infection - Group home | 0.24 |
| Nose bleeding (when “Bleeding (Haemorrhage)” was selected as a symptom prior to SARS-COV-2 testing) | 0.23 |
| Cutaneous bleeding (includes easy bruising) (when “Bleeding (Haemorrhage)” was selected as a symptom prior to SARS-COV-2 testing) | 0.19 |
| Low molecular weight heparin taken prescribed prior to qualifying SARS-CoV-2 test | 0.15 |
| Acetaminophen taken within 24 hours of index visit | 0.14 |
| Ear pain recorded during index ED visit | 0.11 |
| Ever hypotensive in ED (SBP < 100 mm Hg) | 0.1 |
| Risk for COVID-19 infection - Employed, healthcare worker with patient contact | 0.09 |
| Cardiac arrest prior to SARS-COV-2 testing | 0.08 |
| Heart Rate* | 0.08 |
| Race - American Indian or Alaska Native | 0.08 |
| Currently pregnant | 0.2 |
| Vaginal bleeding (when “Bleeding (Haemorrhage)” was selected as a symptom prior to SARS-COV-2 testing) | 0.06 |
| Syncope prior to SARS-COV-2 testing | 0.06 |
| Warfarin taken prescribed prior to qualifying SARS-CoV-2 test | 0.05 |
| Conjunctivitis prior to SARS-COV-2 testing | 0.03 |
| Lymphadenopathy prior to SARS-COV-2 testing | 0.03 |
| Risk for COVID-19 infection - Travel to the USA from a country with known endemic disease | 0.02 |
| Lowest O2 saturation while in ED* | 0.02 |
| Risk for COVID-19 infection - Institutional exposure: assisted living facility | 0.02 |
| Abdominal pain | <0.0001 |
| Ace inhibitors taken within 24 hours of index visit | <0.0001 |
| Age at time of visit * | <0.0001 |
| Angiotensin receptor blocker use | <0.0001 |
| Apixaban taken prescribed prior to qualifying SARS-CoV-2 test | <0.0001 |
| Asthma (prior history) | <0.0001 |
| Atrial fibrillation (prior history or new onset) | <0.0001 |
| BiologicalSex | <0.0001 |
| Bleeding (Haemorrhage)prior to SARS-COV-2 testing | <0.0001 |
| Bloody emesis (when “Bleeding (Haemorrhage)” was selected as a symptom prior to SARS-COV-2 testing) | <0.0001 |
| Body mass index* | <0.0001 |
| Cancer (active or in remission) | <0.0001 |
| Chest pain recorded during index ED visit | <0.0001 |
| Chronic obstructive pulmonary disease (prior history) | <0.0001 |
| Cough without sputum (dry) recorded during index ED visit | <0.0001 |
| Daily alcohol use | <0.0001 |
| Diabetes Mellitus (prior history) | <0.0001 |
| Diarrhea prior to SARS-COV-2 testing | <0.0001 |
| Diastolic Blood Pressure (mm Hg)* | <0.0001 |
| Fatigue / Malaise recorded during index ED visit | <0.0001 |
| Headache prior to SARS-COV-2 testing | <0.0001 |
| Heart failure (either systolic or ejection fraction preserved) | <0.0001 |
| History of fever recorded during index ED visit | <0.0001 |
| Hyperlipidemias (hyperlipidemia of any type, includes currently taking a statin) | <0.0001 |
| Latin Ethnicity | <0.0001 |
| Marijuana use | <0.0001 |
| Medicaid or no insurance | <0.0001 |
| Methamphetamine use | <0.0001 |
| Muscle aches (Myalgia) recorded during index ED visit | <0.0001 |
| No anticoagulants taken prescribed prior to qualifying SARS-CoV-2 test | <0.0001 |
| No known risk for COVID-19 infection | <0.0001 |
| No substance use | <0.0001 |
| Obesity (by diagnosis, provider interpretation or body mass index >35 Kg/m^2) | <0.0001 |
| Olfactory/taste disturbance prior to SARS-COV-2 testing | <0.0001 |
| Opioid dependency | <0.0001 |
| Organ transplantation (any organ) | <0.0001 |
| Other (not specified) antihypertensive medication taken within 24 hours of index visit | <0.0001 |
| Other bleeding site (not specified) (when “Bleeding (Haemorrhage)” was selected as a symptom prior to SARS-COV-2 testing) | <0.0001 |
| Other lung disease (pulmonary fibrosis, cystic fibrosis, bronchiectasis, pulmonary hypertension) | <0.0001 |
| Cocaine use | <0.0001 |
| Injection drug use | <0.0001 |
| Oxygen saturation at triage* | <0.0001 |
| Prior ischemic heart disease (myocardial infarction, or any coronary revascularization including stent or CABG) | <0.0001 |
| Prior venous thromboembolism | <0.0001 |
| Race - Asian | <0.0001 |
| Race - Black or African American | <0.0001 |
| Race - Unknown/Other | <0.0001 |
| Race - White | <0.0001 |
| Recent seizure prior to SARS-COV-2 testing | <0.0001 |
| Respiratory distress on arrival | <0.0001 |
| Respiratory Rate (breaths/min)* | <0.0001 |
| Risk for COVID-19 infection - Caretaker, partner or family member in close contact with a person with known or suspected COVID-19 | <0.0001 |
| Risk for COVID-19 infection - Employed, non-health care worker, contact with family, and/or friends, and/or general public | <0.0001 |
| Risk for COVID-19 infection - Institutional exposure:, nursing home | <0.0001 |
| Risk for COVID-19 infection - Other risk exposure (not specified) | <0.0001 |
| Risk for COVID-19 infection - Person experiencing homelessness | <0.0001 |
| Risk for COVID-19 infection - Sick contacts without confirmed COVID-19 | <0.0001 |
| Risk for COVID-19 infection - Unemployed or retired, social contact with friends, family and/or general public | <0.0001 |
| Rivaroxaban taken prescribed prior to qualifying SARS-CoV-2 test | <0.0001 |
| Runny nose (Rhinorrhoea) recorded during index ED visit | <0.0001 |
| Skin rash | <0.0001 |
| Smoker (Current smoker regardless of number of cigarettes; includes vaping, cigars or pipe smoking) | <0.0001 |
| Sore throat recorded during index ED visit | <0.0001 |
| Statins taken within 24 hours of index visit | <0.0001 |
| Systemic Hypertension (Regardless of treatment status; can also be diagnosed by diastolic BP 110 mm Hg in ED) | <0.0001 |
| Systolic Blood Pressure (mmHg) | <0.0001 |
| Temperature (°C) | <0.0001 |
| Thiazolidinediones taken within 24 hours of index visit | <0.0001 |
| Total number of symptoms | <0.0001 |
| Wheezing recorded during index ED visit | <0.0001 |
